# The role of CD8^+^ T cells in the immunoregulation of osteoporosis

**DOI:** 10.3389/fimmu.2026.1874242

**Published:** 2026-06-12

**Authors:** ZiChao Chen, Hao Hu, Die Hu, QinChuan Du, HuanZhi Yang, RongHua Xu, Peng Yu, XingBo Hu, Jun Hu

**Affiliations:** 1 Organization Kunming Calmette International Hospital, KunMing, China; 2 Organization Xiangnan University, ChenZhou, China; 3 Organization Hunan University of Medicine, HuaiHua, China

**Keywords:** CD8+ T cells, cellular senescence, immunotherapy, interferon-γ, osteoimmunology, osteoporosis

## Abstract

**Introduction:**

Osteoporosis weakens the skeleton by lowering bone mass and damaging bone microarchitecture. A growing body of evidence points to disruptions in osteoimmune homeostasis as a key driver of this disease. In particular, apoptosis of bone cells and immune cells appears to be a critical contributing mechanism. Within the bone marrow, CD8^+^ T cells display a surprising functional duality: whether they protect bone or promote its loss depends heavily on local signals. Under healthy conditions or after mechanical loading, activated CD8^+^ T cells help maintain bone mass. They do so mainly by secreting interferon‑γ (IFN‑γ), which blocks osteoclast formation through interference with NF‑κB and MAPK signaling. However, this protective role is not fixed. Pathological cues such as estrogen deficiency can flip CD8^+^ T cells into a bone‑destroying phenotype. For example, when estrogen drops, muscle‑derived IL‑33 decreases. That drop prompts bone marrow CD8^+^ T cells to release large amounts of CCL5. CCL5 then binds to CCR3 on osteoclast precursors and activates the ERK pathway, thereby accelerating bone loss in postmenopausal osteoporosis. Aging and chronic inflammation add another layer of complexity. They drive the accumulation of senescent CD28^-^ CD8^+^ T cells. These aged cells no longer produce enough IFN‑γ to protect bone; instead, they adopt a senescence‑associated secretory phenotype (SASP) that suppresses osteoblast differentiation and reduces mesenchymal stromal cell viability. Moreover, apoptosis of osteoblasts and osteocytes—triggered by TNF‑α, IFN‑γ, or glucocorticoids—worsens bone loss. New findings suggest that CD8^+^ T cells may fuel this apoptotic process through Fas/FasL interactions and the granzyme/perforin pathway. This review brings together these context‑dependent mechanisms. We place special emphasis on how hormonal shifts, metabolic changes, and inflammatory mediators converge to decide whether CD8^+^ T cells support skeletal integrity or drive bone resorption.

**Methods:**

We performed a narrative review of recent studies on CD8^+^ T cells in osteoporosis. We searched PubMed and Web of Science using keywords such as “osteoporosis”, “osteoimmunology”, “CD8^+^ T cells”, “IFN‑γ”, “CCL5”, “cellular senescence”, “SASP”, “Fas/FasL”, and “granzyme”. From the retrieved articles, we selected original research (including *in vitro* co‑culture systems, animal models, and clinical samples) and relevant reviews. We then extracted and integrated key mechanistic evidence regarding the dual functionality of CD8^+^ T cells and its regulatory pathways.

**Results:**

CD8^+^ T cells have two opposing functions. Under healthy conditions or mechanical loading, activated CD8^+^ T cells secrete IFN‑γ, which inhibits osteoclast formation by interfering with NF‑κB and MAPK signaling, thus maintaining bone mass. In contrast, when estrogen is deficient, reduced muscle‑derived IL‑33 causes bone marrow CD8^+^ T cells to release large amounts of CCL5. CCL5 binds to CCR3 on osteoclast precursors and activates the ERK pathway, accelerating bone loss. Aging and chronic inflammation shift the balance. Aging and chronic inflammation lead to the accumulation of senescent CD28^-^ CD8^+^ T cells. These cells no longer produce sufficient protective IFN‑γ. Instead, they exhibit a senescence‑associated secretory phenotype (SASP) that suppresses osteoblast differentiation and reduces mesenchymal stromal cell viability. CD8^+^ T cells may promote bone cell apoptosis through multiple pathways. TNF‑α, IFN‑γ, or glucocorticoids can trigger apoptosis of osteoblasts and osteocytes. Newer evidence suggests that CD8^+^ T cells amplify this process via Fas/FasL interactions and the granzyme/perforin pathway. Two newly identified CD8^+^ T cell‑derived molecules deserve attention. Granzyme K protects against bone loss, whereas CCL5 promotes bone loss. These two molecules offer fresh opportunities for biomarkers and therapies.

**Discussion:**

Our synthesis shows that CD8^+^ T cells act as a double‑edged sword in osteoporosis: their functional switch depends on the integration of hormonal, metabolic, and inflammatory signals. Under normal conditions or after mechanical loading, the anti‑osteoclastogenic effect of IFN‑γ dominates. But when estrogen levels fall, the IL‑33/CCL5/CCR3/ERK axis becomes activated, turning CD8^+^ T cells toward a bone‑resorbing phenotype. During aging, the buildup of CD28⁻ CD8^+^ T cells and their SASP further weakens bone formation and worsens bone cell death through apoptotic pathways. These observations point to a more precise therapeutic strategy. Instead of broadly modulating T cells, one could specifically target pathogenic pathways—for example, by interfering with the IL 33/ST2/CCL5 cascade or by clearing immune senescent CD28⁻ subsets. Such approaches might help restore osteoimmune balance. Two recently discovered CD8^+^ T cell derived molecules, granzyme K and CCL5, are particularly interesting because they respectively protect against and promote bone loss. They could serve as novel biomarkers or even therapeutic targets. Future studies should examine the cell type specific regulation of these pathways in vivo and test the safety and efficacy of targeted interventions.

## 1 Introduction

Osteoporosis is the most prevalent skeletal disease worldwide, and the fractures it causes severely impair the health and quality of life of middle-aged and older adults (1, 2). The disorder has traditionally been attributed to hormonal changes (particularly estrogen deficiency), nutritional deficits, and genetic susceptibility (3). The emergence of osteoimmunology, however, has shown that a complex, bidirectional regulatory network intimately links the immune and skeletal systems (4, 5). T lymphocytes, central players in adaptive immunity, are now known to participate extensively in the control of bone metabolism (6). The role of CD8^+^ T cells (cytotoxic T cells) has become a particular focus within this field (4, 7). Conditions such as estrogen withdrawal, aging, chronic inflammation, or the use of drugs like glucocorticoids can markedly alter the abundance, phenotype, function, and cytokine secretion profile of CD8^+^ T cells. These alterations then influence osteoblast differentiation and activity, as well as osteoclast formation and function. The net effect is a disruption of bone homeostasis (8). For example, activated CD8^+^ T cells can secrete IFN-γ and thereby suppress osteoclastogenesis (9, 10). Yet, they may also release chemokines, including CCL5, that drive osteoclast formation. In osteolytic diseases, senescent or prematurely aged T cells (a population that includes some CD8^+^CD28⁻ cells) are thought to acquire pro-resorptive properties (8). A clearer picture of how CD8^+^ T cells operate in osteoporosis is therefore needed. Such knowledge will help to explain the immunological basis of the disease, identify new biomarkers, and guide the design of targeted therapies (11, 12).

Clinical studies further support a link between CD8^+^ T cells and bone mineral density (BMD). In a retrospective analysis of older adults with osteoporosis, the absolute count of peripheral blood CD8^+^ T lymphocytes was significantly lower in osteoporotic patients than in non-osteoporotic controls, and BMD at the right femoral neck declined in parallel with the reduction in CD8^+^ T-cell numbers (13). Another study of an elderly cohort identified a low total lymphocyte count as an independent risk factor for BMD loss (14). These observations suggest that a decrease in CD8^+^ T lymphocytes may contribute to the bone metabolic dysfunction that accompanies immunosenescence (13). In a model of estrogen-deficiency-induced osteoporosis, wheel-running exercise activated CD8^+^ T cells and restored their expression of IFN-γ. This suppression of NF-κB and MAPK signaling inhibited osteoclastogenesis and attenuated bone loss, and it points to a key immunoregulatory function for CD8^+^ T cells in the osteoprotective effects of exercise (9). The functional state of CD8^+^ T cells is equally important. CD8^+^ T cells can secrete the osteoanabolic factor Wnt-10b under certain conditions, thereby promoting bone formation (15). Conversely, accumulation of senescent or functionally exhausted CD8^+^CD28⁻ T-cell subsets correlates with bone loss in several pathological settings (8, 16). Changes in the abundance, subset composition, and activation status of CD8^+^ T cells therefore represent key immunological determinants of bone homeostasis, reflecting the multiple and context-dependent roles these cells play in the skeleton.

## 2 CD8^+^ T cells in physiological bone remodeling and homeostasis

### 2.1 CD8^+^ T cell-derived IFN-γ suppresses osteoclastogenesis

The bone marrow microenvironment contains a resident population of CD8^+^ T cells that can, under certain conditions, regulate bone remodeling through the secretion of interferon-γ (IFN-γ) (10). IFN-γ acts directly on osteoclast precursors and inhibits their differentiation by interfering with the classical NF-κB and MAPK signaling pathways (9). This inhibitory effect is both dose- and time-dependent, a feature that demonstrates the central role of IFN-γ in restraining bone resorption (9). Animal experiments have confirmed the importance of the CD8^+^ T cell–IFN-γ axis. In ovariectomized (OVX) mice, a model of postmenopausal osteoporosis, wheel-running exercise increased the proportion of CD8^+^ T cells in both the spleen and the bone marrow and restored their expression of IFN-γ (9). The exercise-induced rise in CD8^+^ T-cell-derived IFN-γ subsequently suppressed osteoclastogenesis and prevented bone loss in OVX mice (9). CD8^+^ T cells, via their production of IFN-γ, thus act as a critical brake on bone resorption and serve as an essential link between the immune and skeletal systems in the maintenance of bone balance.

### 2.2 Homeostatic crosstalk between CD8^+^ T cells and other osteoimmune cells

Within the complex immune microenvironment of the bone marrow, CD8^+^ T cells do not act in isolation. They form part of a dynamic, balanced network that also includes CD4^+^ T cells, regulatory T cells (Treg), B cells, and innate immune cells, and together these populations govern bone homeostasis (17). CD8^+^ T cells can influence the function of other immune cells through their particular cytokine secretion profile, thereby modulating bone metabolism indirectly. In the bone marrow of mice bearing non-metastatic 67NR breast tumors, for example, CD8^+^ T cells establish an anti-osteoclastogenic milieu rich in IFN-γ and interleukin-10 (IL-10) while producing only low levels of receptor activator of nuclear factor-κB ligand (RANKL) (17). This distinctive cytokine environment inhibits osteoclastogenesis directly and may also act by shaping the function of CD4^+^ T-cell subsets. CD8^+^ T cells from 67NR tumors can, both *in vitro* and *in vivo*, suppress the phenotype of CD4^+^ T cells specific for metastatic 4T1 tumors; this suppression may contribute to the preservation of trabecular bone mass (17). CD8^+^ T cells also engage in close interactions with Treg cells. Bone marrow-derived CD8^+^FoxP3^+^ regulatory T cells, which are potent inhibitors of osteoclastogenesis, are present at increased frequencies in 67NR tumor-bearing mice (17). Although the underlying mechanisms are not fully resolved, CD8^+^ T cells may support Treg function through the secretion of factors such as IL-10, helping to maintain a Th17/Treg balance that favors bone formation (18). In a xenotransplantation model that used cancellous bone from α1,3-galactosyltransferase-knockout (GTKO) pigs to repair bone defects, a decrease in the ratio of CD4^+^ to CD8^+^ T cells was observed, along with suppressed levels of IFN-γ and IL-2. These changes attenuated graft rejection and facilitated new bone formation (19). The finding indicates that shifts in the proportion and function of CD8^+^ T cells can alter the overall immune response and, in turn, influence skeletal repair outcomes (20). Direct evidence for interactions between CD8^+^ T cells and mesenchymal stromal cells (MSCs) remains limited in the current literature. Even so, by modulating the inflammatory state and cytokine networks within the bone marrow niche, CD8^+^ T cells are well positioned to affect indirectly the differentiation of MSCs toward an osteoblast lineage. Whether this indirect regulation occurs *in vivo* is an open question that merits further study.

## 3 CD8^+^ T cells in the pathogenesis of postmenopausal osteoporosis

### 3.1 Estrogen deficiency alters CD8^+^ T-cell activation and function

In postmenopausal osteoporosis (PMOP), estrogen deficiency creates a pro-inflammatory immune environment. It shifts the resting Th2/Tc2 balance toward a Th1/Tc1 phenotype, and CD8^+^ T cells start producing more IFN-γ and TNF-α. Two CD8^+^ T-cell-related pathways have recently emerged as important players in PMOP. One involves CD8a^+^GZMK^+^ T cells. They secrete granzyme K (GzmK), which then suppresses osteoclastogenesis through the p38-MAPK pathway—a compensatory protective response (12). The other is the IL-33–ST2–CCL5–CCR3 axis, which links muscle, bone, and immunity. During sarcopenia, muscle-derived IL-33 drops. That drop releases CD8^+^ T cells from normal inhibition, so they overproduce CCL5. CCL5 in turn activates ERK signaling in osteoclast precursors and drives their differentiation (21). Estrogen deficiency is the central driver of postmenopausal osteoporosis (PMOP), and its effects on the immune system, particularly on CD8^+^ T cells, is a growing focus (22). Work with the ovariectomized (OVX) mouse, a classic model of the postmenopausal state, indicates that estrogen loss markedly changes the distribution and activation status of CD8^+^ T cells. In OVX mice, the proportion and absolute number of CD8^+^ T cells increase significantly in both the spleen and the bone marrow, and activation markers such as CD69 are upregulated. These changes suggest a systemic expansion and activation of CD8^+^ T cells driven by estrogen deficiency (23), a shift linked to a chronic low-grade inflammatory phenotype that primes the disruption of osteoimmune balance (24, 25). T cells express estrogen receptors (ER), and estrogen deficiency directly impairs ER signaling within CD8^+^ T cells (26). Studies of double-negative T cells (DNT cells, defined as CD3^+^TCRαβ^+^CD4⁻CD8⁻NK1.1⁻) show that OVX mice have a reduced frequency and immunoregulatory capacity of DNT cells, along with increased apoptosis and suppressed proliferation (27). Transcriptomic analysis further confirms downregulation of the ER signaling pathway and aberrant activation of the nuclear factor-κB (NF-κB) pathway in DNT cells after estrogen loss. *In vitro*, estrogen stimulation enhances DNT cell immunoregulatory function, upregulates estrogen receptor Esr1, and inhibits NF-κB activation (27). These events weaken ER signaling and activate NF-κB, compromising the survival and function of regulatory CD8^+^ T-cell subsets such as DNT cells. They may also push other CD8^+^ T-cell subsets toward a pro-inflammatory phenotype, enhancing cytotoxicity or the secretion of pro-inflammatory cytokines. The net result is an amplification of immune dysregulation and bone destruction in PMOP. IL-33 is often described as an alarmin, but that label sells it short. Inside cells, it can also regulate gene transcription. Once released, it binds to ST2 receptors on various bone-related cells, and the effects differ markedly by cell type. On CD8+ T cells*, as discussed above, IL-33 suppresses pathogenic CCL5 secretion. This indirect effect inhibits osteoclastogenesis (28). However, IL-33 does not need T cells to influence bone. On osteoclast precursors, high concentrations of IL-33 can directly activate NF-κB and MAPK signaling through ST2, which paradoxically *promotes* osteoclast differentiation. Therefore, the same cytokine can tilt the balance either way, depending on its concentration and local context. On osteoblasts, the story takes another turn. Recent work, including a study on age-related bone loss (21), shows that IL-33 directly stimulates osteoblast differentiation and mineralization. It does so by enhancing Runx2 and Osterix expression while protecting osteoblasts from oxidative stress-induced apoptosis. This anabolic effect matters especially for senile osteoporosis, where IL-33 levels drop with age. On mesenchymal stromal cells (MSCs), IL-33 promotes cell survival and osteogenic commitment. Taken together, IL-33 is not simply a T-cell modulator. It is a multi-tissue regulator that connects muscle function, immune status, and skeletal health. Any therapeutic strategy based on IL-33—for example, recombinant IL-33 or ST2 agonists—will need to account for these divergent effects.

### 3.2 The pro-osteoclastogenic role of CD8^+^ T-cell-derived CCL5 in PMOP

In the setting of postmenopausal osteoporosis, skeletal muscle atrophy or impaired muscle function reduces the secretion of myokines such as IL-33, a change that affects the bone marrow microenvironment via the muscle–bone axis (29). In ovariectomized (OVX) mice, a model of PMOP, bone marrow CD8^+^ T cells occupy a central position in this pathway. These cells express ST2, the receptor for IL-33, on their surface, and a decline in muscle-derived IL-33 signaling triggers a shift in CD8^+^ T-cell function (28). Under these conditions, bone marrow CD8^+^ T cells markedly increase their secretion of the chemokine CCL5 (28). CCL5 then binds to the specific receptor CCR3 on the surface of osteoclast precursors (30). This binding event activates downstream signaling pathways, most notably the extracellular signal-regulated kinase (ERK) pathway (28). Activation of ERK signaling directly stimulates the differentiation of osteoclast precursors into mature osteoclasts and enhances the bone-resorbing activity of existing osteoclasts (31). The sequence that begins with muscle atrophy and proceeds through CD8^+^ T cell-mediated CCL5 secretion thus constitutes a pathological route that directly accelerates bone resorption. This route places CD8^+^ T cells at the interface of muscle–bone crosstalk: they convert a signal that reflects the functional state of peripheral muscle (transmitted via the IL-33/ST2 axis) into a local pro-osteoclastic signal within the bone marrow (mediated by the CCL5/CCR3/ERK axis). On a background of estrogen deficiency, this additional input drives and amplifies the bone loss seen in OVX mice (32). Interrupting this axis, either at the level of CD8^+^ T cells or their secreted CCL5, may therefore provide a new strategy to counteract bone loss in PMOP.

## 4 Roles of aged and prematurely senescent CD8^+^ T cells in osteoporosis

### 4.1 Accumulation and characteristics of CD8^+^CD28⁻ senescent T cells

Immunosenescence accompanies physiological aging and chronic inflammatory disease. One hallmark is the expansion of T-cell subsets that have lost the co-stimulatory molecule CD28 (CD28⁻), a shift that is especially evident within the CD8^+^ T-cell compartment (8). CD8^+^CD28⁻ T cells are viewed as terminally differentiated, senescent-like, or prematurely aged cells. They show poor proliferative capacity, shortened telomeres, an altered secretory profile, and a pro-inflammatory senescence-associated secretory phenotype (SASP) (33). Co-expression of TIGIT and Helios defines immunosenescent CD8^+^ T cells with greater precision; these cells fail to proliferate upon stimulation *in vitro* and have impaired induction of the activation markers CD69 and CD25 (33). With advancing age, CD8^+^CD28⁻ T cells accumulate markedly in peripheral blood, and the extent of this accumulation tracks closely with chronological age (34). Chronic viral infections, cytomegalovirus (CMV) infection in particular, drive CD8^+^ T cells toward a terminally differentiated phenotype (e.g., CD27⁻CD28⁻) and hasten their senescence (35). Frequencies of CD8^+^CD28⁻ or CD8^+^CD57^+^ T cells are elevated in the peripheral blood of patients with several diseases, including acute myeloid leukemia (AML), non-small cell lung cancer (NSCLC), and multiple myeloma (MM) (36–38). These cells typically exhibit impaired proliferation, shortened telomeres, and heightened expression of cytotoxic molecules such as granzyme B and perforin (37). Direct evidence in osteoporosis is still sparse, but a comparable process is likely at work. T lymphocytes with a prematurely aged phenotype, marked by the absence of CD28, accumulate in osteolytic disorders, osteoporosis among them (8, 39). An increased proportion of senescent CD8^+^CD28⁻ T cells within the bone microenvironment is therefore likely to correlate inversely with bone mineral density (BMD) and to contribute to the pathogenesis of osteoporosis.

### 4.2 Bone-destructive mechanisms of senescent CD8^+^ T cells

Senescent CD8^+^CD28⁻ T cells lose the osteoclast-suppressive capacity of normal CD8^+^ T cells, for instance, a decline in IFN-γ secretion, and instead drive bone resorption directly through the release of pro-inflammatory and osteoclastogenic factors such as tumor necrosis factor-α (TNF-α) and RANKL (8, 40). In osteolytic diseases, prematurely senescent CD4^+^CD28⁻ T lymphocytes are a known source of elevated osteoclastogenic mediators, including TNF-α and RANKL (8, 41). Direct evidence for equivalent production by CD8^+^CD28⁻ T cells is more limited, but senescent T cells overall shift toward a pro-inflammatory phenotype (42). These cells also secrete cytotoxic molecules, including granzymes, that inhibit osteoblast differentiation and compromise mesenchymal stromal cell (MSC) survival. Bone loss therefore proceeds from both impaired bone formation and enhanced resorption (8). A negative correlation between prematurely senescent CD8^+^CD28⁻ T lymphocytes and bone healing or regeneration is well documented, and the underlying mechanisms likely involve suppressed osteoblast differentiation and diminished MSC viability (8). High expression of granzyme B and perforin endows these cells with considerable cytotoxic potential, which may directly injure bone-forming cells (37). The inflammatory milieu generated by senescent CD8^+^ T cells also activates neighboring immune cells, notably macrophages, in a positive feedback loop that magnifies bone destruction. In rheumatoid arthritis synovial fluid, for example, activated effector memory T lymphocytes (CD8^+^ T cells included) pair with neutrophil–dendritic cell hybrids (N-DCs), an interaction that may exacerbate local inflammation and tissue injury. Lactate that accumulates in the tumor microenvironment suppresses TCR/CD28-induced CD8^+^ T-cell activation through the MondoA–TXNIP axis, fostering an immunosuppressive niche (43). A parallel sequence of events could occur in bone. Senescent CD8^+^ T cells release pro-inflammatory cytokines (such as TNF-α and IL-6) via their SASP. These factors directly stimulate osteoclastogenesis while also recruiting macrophages and polarizing them toward an M1 phenotype. The activated macrophages then secrete further osteoclastogenic mediators and intensify the inflammatory response. This self-reinforcing loop perpetuates bone resorption and curtails bone formation.

### 4.3 Senile osteoporosis: when CD8+ T cells age badly

Chronological aging causes bone loss through mechanisms that partly overlap with, yet differ from, postmenopausal osteoporosis. Hormonal decline plays a lesser role here; instead, the aging immune system—and CD8+ T cells in particular—undergoes profound changes. Immunosenescence does not simply weaken immunity. It also turns formerly protective CD8+ T cells into drivers of bone fragility. One hallmark of this process is the accumulation of CD28⁻CD8^+^ T cells. These cells are terminally differentiated, often carry CD57, and have lost the co-stimulatory receptor CD28. They do not just fail to protect bone; they actively harm it. Unlike their CD28^+^ counterparts, senescent CD8^+^ T cells secrete a senescence-associated secretory phenotype (SASP) rich in IL-6, TNF-α, and RANKL. At the same time, they lose the capacity to produce enough IFN-γ to restrain osteoclasts (8). The net result shifts the bone microenvironment toward resorption. Beyond promoting osteoclast formation, these senescent cells directly suppress bone formation. Granzyme B and perforin, which they release in large amounts, induce apoptosis in osteoblasts and mesenchymal stromal cells (37). This dual attack—more resorption plus less formation—explains why aged bone heals poorly and fractures easily.

Metabolic dysfunction within aged CD8+ T cells amplifies the problem. They show altered nutrient sensing (for instance, through the MondoA-TXNIP axis) and impaired mitochondrial respiration. This metabolic profile pushes them toward a glycolytic, pro-inflammatory state, feeding a cycle of inflammaging that accelerates bone loss (43, 44). Therapies that restore mitochondrial health or clear senescent CD8+ T cells (senolytics) might therefore break this cycle (**Table 1**).

**Table 1:** Key signaling pathways involving CD8^+^ T cells in bone regulation.

Signaling axis/pathway	Upstream trigger	Downstream effectors	Target cell	Effect on bone	Refs
IFN-γ–TRAF6–NF-κB/MAPK	IFN-γ from CD8^+^ T cells/TcREG	TRAF6 degradation → ↓NF-κB, JNK	Osteoclast precursors	Inhibition of osteoclast differentiation	(9, 10)
CD8a^+^GZMK^+^ T cell–p38/MAPK	GzmK from CD8a^+^GZMK^+^ T cells	↓p38-MAPK phosphorylation → ↓NFATC1	Osteoclast precursors	Inhibition of osteoclastogenesis	(12)
IL-33–ST2–CCL5–CCR3–ERK	Muscle atrophy (↓IL-33) → ↑CCL5 from CD8^+^ T cells	CCL5/CCR3 → ERK activation	Osteoclast precursors	Promotion of osteoclastogenesis	(28)
RANKL–RANK–TRAF6–NF-κB	Bone marrow T cell RANKL (GIOP)	TRAF6 → NF-κB	Osteoclast precursors	Promotion of osteoclast formation	(57)
Wnt-10b–β-catenin	cAMP-activated CD8^+^ T cells	Canonical Wnt signaling	Osteoblast precursors	Promotion of bone formation	(15)
ERS–UPR–sXBP1–THBS2	ER stress → UPR	sXBP1 inhibits THBS2 → relief of AKT-mTOR inhibition → ↑CD8^+^ T-cell proliferation	CD8^+^ T cells (CD36^+^)	Enhanced cytotoxicity (to be validated in OP)	(49)
IFN-γ–RANKL cross-regulation	CD8^+^ T-cell activation	IFN-γ interferes RANKL–RANK → TRAF6 degradation	Osteoclast precursors	Negative regulation of resorption	(10)

↑, Increase, upregulation, or elevated level; ↓, Decrease, downregulation, or reduced level; →, Leads to, results in, or triggers (e.g., “IFN‑γ → ↓NF‑κB/MAPK”).

## 5 CD8^+^ T cells in the context of energy metabolism and endoplasmic reticulum stress-associated osteoporosis

### 5.1 Energy metabolism-related genes and their association with CD8^+^ T-cell infiltration

Bioinformatic analyses have identified notable differences in immune cell infiltration patterns among osteoporosis patients, particularly between groups with distinct bone mineral density (BMD) values. A study based on the GSE56814,GSE62402 and GSE7158 datasets found that peripheral blood samples from individuals with high BMD differed significantly from those with low BMD across multiple immune cell types (45). The proportions of central memory CD8^+^T cells, effector memory CD8^+^T cells, and activated CD8^+^T cells showed statistically significant variation between the high and low BMD groups (45). This observation shows that changes in BMD are closely tied to systemic immune status, especially the distribution of CD8^+^T cell subsets. Further analysis identified 72 differentially expressed energy metabolism-related genes (DE-EMGs) and established a diagnostic model that includes B4GALT4, ADH4, ACAD11, B4GALT2, and PPP1R3C (45). The expression of B4GALT4, a key gene in this model, correlated positively with the degree of CD8^+^T-cell infiltration (45). This implies that reprogramming of energy metabolism may directly affect the recruitment, activation, or functional state of CD8^+^T cells within the skeletal microenvironment. Similar associations occur in other diseases. In renal cell carcinoma, CD8^+^T-cell infiltration into the tumor microenvironment is linked to specific metabolic reprogramming events, such as the downregulation of oxidative phosphorylation (46). In osteosarcoma, the expression of the mitochondrial-related genes UCP2 and PRDX4 also tracks closely with the extent of CD8^+^T-cell infiltration (47). These findings support the idea of a “metabolism–immunity–skeleton” axis. Altered metabolic states in bone cells or immune cells may serve as upstream events that modify CD8^+^T-cell function and thereby contribute to osteoporosis. For instance, aberrant expression of energy metabolism genes could change the local availability of metabolic substrates such as glutamine, indirectly influencing the activity and proliferation of CD8^+^T cells (48). Clarifying how energy metabolism-related genes govern CD8^+^T-cell behavior will advance the immunological understanding of osteoporosis and inform the design of new therapies.

### 5.2 Endoplasmic reticulum stress contributes to bone loss via effects on CD8^+^ T cells

Work from several groups points to endoplasmic reticulum stress (ERS) and the resultant unfolded protein response (UPR) as important drivers of osteoimmune dysregulation in osteoporosis. Oncology research offers a helpful analogy for how such a connection might operate. In head and neck squamous cell carcinoma, interference with the tricarboxylic acid cycle triggers ERS and engages the UPR sensor IRE1α, which in turn drives up the expression of spliced X-box binding protein 1 (sXBP1) (49). Acting as a transcription factor, sXBP1 suppresses THBS2 (thrombospondin-2) transcription. The fall in THBS2 protein weakens the normal brake on AKT–mTOR signaling within CD36^+^CD8^+^ tumor-infiltrating T cells, and the net effect is a boost in both their proliferation and cytotoxic capacity (49). Although this exact sequence of molecular events has not been confirmed in skeletal tissue, it establishes a more general concept: ERS, acting via UPR-driven transcriptional changes, can alter the local signaling milieu that shapes T-cell behavior. A comparable chain of events probably unfolds in the osteoporotic bone microenvironment. Conditions such as estrogen deprivation or mechanical unloading are known to impose ERS on resident bone cells, including osteoblasts and osteoclasts, and to activate the UPR. This stress response may modify the local collection of cytokines or alter the surface display of immunomodulatory proteins (PD-L1, for example) in ways that steer infiltrating CD8^+^ T cells toward a pro-inflammatory or cytotoxic state (50). These activated T cells could then aggravate the disruption of bone metabolism, chiefly by releasing larger amounts of interferon-γ (IFN-γ) and other factors that promote osteoclast formation and bone resorption. ERS is therefore more than a mechanism for coping with misfolded proteins; it may also act as a central junction where metabolic disturbance, remodeling of the immune niche, and pathological bone loss converge (51). Strategies aimed at dampening ERS or at modulating specific branches of the UPR might, for this reason, provide a new way to restrain pathogenic CD8^+^ T-cell activity and slow the progression of osteoporosis.

### 5.3 Apoptosis as a key mechanism in osteoporosis: interplay with CD8^+^ T cells

**Figure 1:**
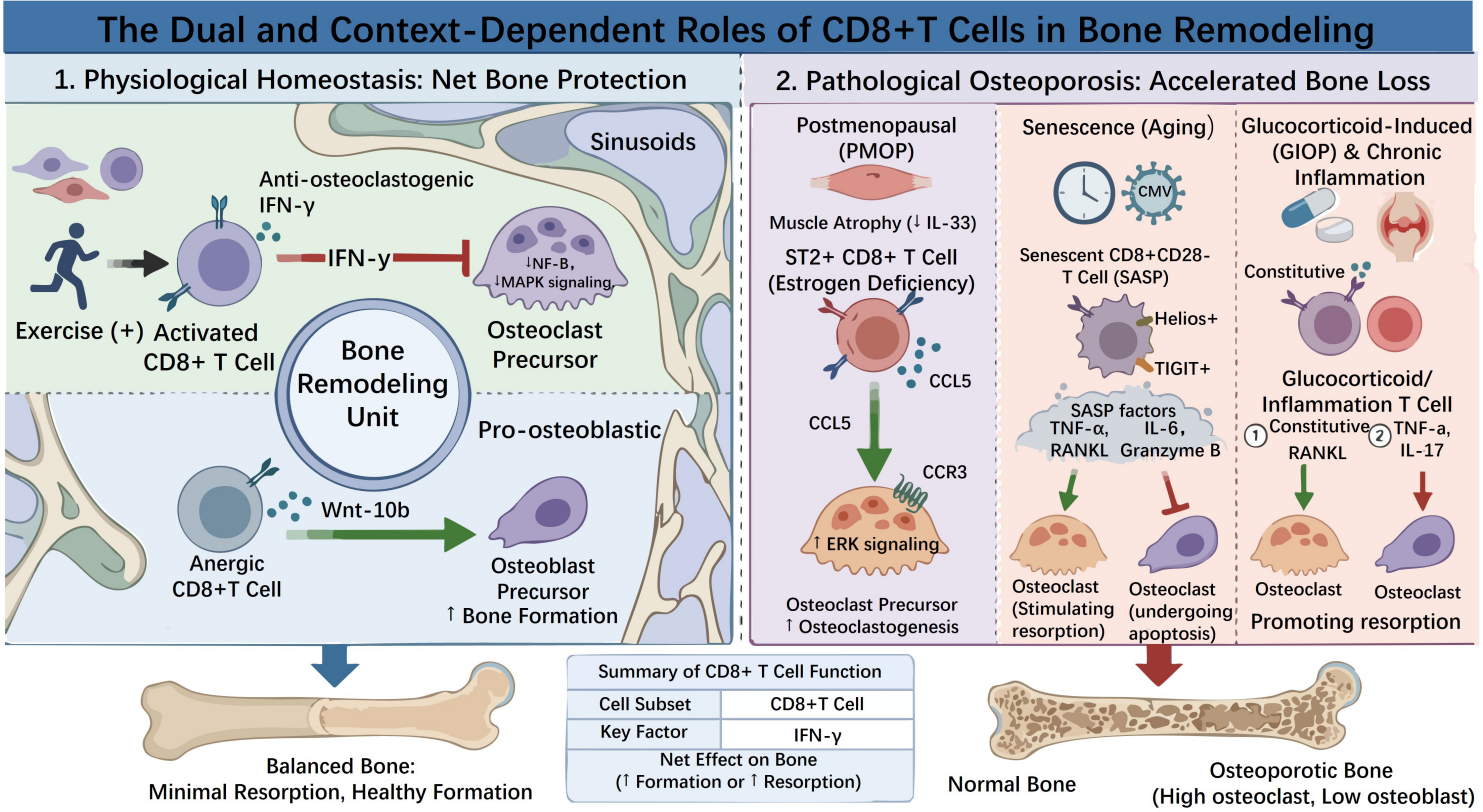
Immunoregulatory mechanisms of CD8^+^ T cells in osteoporosis: from bone protection to bone destruction.

Osteoporosis involves not only reduced bone formation and increased resorption but also excessive death of bone cells. Apoptosis of osteoblasts and osteocytes directly cuts down bone formation and weakens the bone matrix (1). Interestingly, CD8^+^ T cells can both trigger apoptosis and be affected by it within the bone microenvironment. Activated CD8^+^ T cells carry FasL (CD95L) and secrete granzyme B and perforin—molecules best known for killing infected or malignant cells. These same mediators can kill osteoblasts and mesenchymal stromal cells (MSCs). For example, when cocultured with CD8^+^CD28⁻ senescent T cells (which express high levels of granzyme B), osteoblast viability drops sharply through a caspase-dependent process (8). Similarly, IFN-γ from CD8^+^ T cells can boost Fas expression on osteoblasts, making them easy targets for FasL-mediated killing. On the flip side, dying osteocytes and osteoblasts release “find-me” and “eat-me” signals (such as ATP and CX3CL1) that attract and modulate CD8^+^ T cells. This can set up a vicious cycle: inflammation fuels more T-cell activation, which in turn kills more bone-forming cells. What does this mean for therapy? Drugs that reduce apoptosis—like bisphosphonates—are already used in osteoporosis. Whether part of their benefit comes from suppressing pro-apoptotic CD8^+^ T cells remains unknown (52). Senolytic drugs that clear senescent CD8^+^CD28⁻ T cells might do double duty: they reduce SASP factors and also lower the apoptotic load on bone. Future work should map exactly how CD8^+^ T-cell activation leads to osteoblast and osteocyte death. That knowledge could open up new drug targets. Apoptosis is not the only form of programmed cell death that contributes to bone loss, a point worth keeping in mind for future studies. Necroptosis, a regulated caspase-independent cell death pathway mediated by RIPK1/RIPK3/MLKL signaling, has recently been shown to contribute substantially to osteocyte death and bone loss in postmenopausal osteoporosis models (53, 54). Direct comparison in OVX-induced osteoporosis indicated that necroptosis may affect osteocyte death even more than apoptosis (53). Within the inflammatory osteoporotic microenvironment, CD8^+^ T cells can provide TNF-α and IFN-γ, two potent inducers of necroptosis in bone cells (55, 56). Given this, it remains unclear whether CD8^+^ T cells promote bone cell death and subsequent bone loss through necroptotic pathways in addition to classical apoptosis; this question deserves further investigation. From a therapeutic perspective, specific inhibitors of necroptosis such as Nec-1 (targeting RIPK1) have already shown efficacy in attenuating bone loss in OVX animals. This raises the possibility that combining or sequentially targeting both apoptosis and necroptosis could offer superior protection against osteoporosis (53) (**Table 2**; **Figure 1**).

**Table 2:** Phenotypic and functional comparison of CD8^+^ T cells in distinct conditions.

Condition	CD8^+^ T-cell subset	Key molecular alterations	Net effect on bone metabolism	Refs
Physiological	Activated CD8^+^ T cells	IFN-γ ↑	Inhibition of osteoclastogenesis	(9, 10)
Physiological	cAMP-activated CD8^+^ T cells	Wnt-10b ↑	Promotion of bone formation	(15)
Physiological	TcREG (CD8^+^FoxP3^+^)	IFN-γ ↑; T-bet/Eomes^+^	Inhibition of osteoclastogenesis	(10)
PMOP	Bone marrow ST2^+^ CD8^+^ T cells	CCL5 ↑ (IL-33/ST2 axis)	Promotion of osteoclastogenesis	(28)
PMOP	CD8a^+^GZMK^+^ T cells	GzmK ↑, GZMA ↓	Inhibition of osteoclastogenesis	(12)
Aging	CD8^+^CD28⁻ senescent T cells	CD28 loss; ↓ proliferation	Inhibition of osteogenesis (↓ MSC survival)	(8)
GIOP	Bone marrow T cells	RANKL ↑ (constitutive)	Promotion of osteoclastogenesis	(57)
GIOP	Peripheral T cells	↓ number; functional suppression	Impaired osteoimmune surveillance	(60)
Chronic inflammation	Activated effector CD8^+^ T cells	TNF-α ↑, IL-17 ↑	Promotion of bone resorption	(60)
Chronic inflammation	Effector memory CD8^+^ T cells (KLRB1^+^IL7R^+^)	Enhanced cytotoxicity (Tc2/Tc17)	Induction of tissue damage/fibrosis	(79)

↑, Increase, higher expression, or enhanced function; ↓, Decrease, lower expression, or impaired function.

## 6 CD8^+^ T cells in other forms of osteoporosis and comorbid conditions

### 6.1 Changes in CD8^+^ T cells during glucocorticoid-induced osteoporosis

Long-term glucocorticoid therapy is a major cause of secondary osteoporosis. Its broad immunosuppressive effects markedly alter osteoimmune homeostasis (58, 59). Glucocorticoids suppress the proliferation of peripheral blood mononuclear cells (PBMCs) and other immune cells while shifting the proportions and functions of T-cell subsets (60, 61). These changes are central to osteoimmunology and, more specifically, to the concept of “immunoporosis,” a framework that highlights the importance of immune cells, especially T lymphocytes, for skeletal health (60, 62). Work in this area has traditionally emphasized CD4^+^ T cells, but the cytotoxic CD8^+^ T-cell compartment also deserves closer scrutiny (63, 64). The immune system undergoes age-related remodeling marked by a progressive decline in the absolute number of total T cells (CD3^+^), a decline that affects both CD4^+^ and CD8^+^ subsets (65, 66). Extended exposure to glucocorticoids may hasten a similar process, triggering apoptosis or functional suppression of T cells, including CD8^+^ T cells, and thereby weakening osteoimmune surveillance This loss of function could reflect a replicative senescence phenotype. It is typified by an expansion of CD28⁻ T cells, which have shortened telomeres, poor proliferative ability, and an increased output of pro-inflammatory cytokines (65, 67). In clinical settings where traditional Chinese medicine has been used to lower glucocorticoid doses, shifts in CD8^+^ T-cell percentages may be less obvious than those in CD4^+^ T cells. Even so, the combined regimen changes the overall pattern of cytokine secretion (60, 68). A rise in cytokines such as interleukin-6 (IL-6), for instance, might indirectly influence bone metabolism by reshaping the osteoimmune microenvironment (60, 69, 70). In aged individuals, the fraction of CD8^+^ T cells that produce type 1 (IFN-γ, TNF-α) and type 2 (IL-6) cytokines increases with age, particularly within effector/cytotoxic and memory subsets (65). Whether glucocorticoids affect bone remodeling by modulating this cytokine secretory capacity of CD8^+^ T cells is still unclear. Glucocorticoids may therefore disrupt osteoimmune balance and contribute to osteoporosis by directly lowering CD8^+^ T-cell numbers, by altering their functional state (for example, by favoring a pro-inflammatory phenotype), or by accelerating their immunosenescence (57). The specific consequences of glucocorticoids for CD8^+^ T-cell function, and the precise mechanisms that drive glucocorticoid-induced osteoporosis (GIOP), require additional investigation (60). The osteoimmune signature of GIOP is distinct from PMOP: it is characterized by global T-cell lymphopenia with paradoxical, constitutive RANKL expression in the surviving CD8+ T cells. Unlike the inflammatory activation seen in PMOP, glucocorticoids suppress TCR-driven cytokine production but prolong the survival of CD8+ T cells that constitutively express RANKL (51). This allows these cells to promote osteoclast formation without requiring a specific antigenic trigger. A recently described phenomenon in GIOP is the accelerated induction of CD28⁻ senescence-like phenotype in CD8+ T cells, which further amplifies RANKL production and reduces IFN-γ-mediated osteoprotection.

### 6.2 CD8^+^ T cells in osteoporosis associated with inflammatory diseases

Patients with chronic inflammatory conditions such as rheumatoid arthritis and Crohn’s disease have a substantially higher risk of osteoporosis, illustrating the tight link between persistent inflammation and bone loss (71, 72). Bioinformatic analyses have offered a fresh perspective on this comorbidity. Shared diagnostic genes have been identified whose expression correlates negatively with the extent of activated CD8^+^ T-cell infiltration, a finding that indicates the immune infiltration status of CD8^+^ T cells could be a key connection between inflammation and bone metabolism (73, 74). The chronic inflammatory state itself drives sustained immune activation. This activation expands and stimulates T lymphocyte subsets, CD8^+^ T cells among them (60). Activated CD8^+^ T cells can then migrate into skeletal sites or joints. There, they promote osteoclastogenesis and bone resorption, both locally and systemically, by secreting potent pro-inflammatory cytokines such as tumor necrosis factor-α (TNF-α) and interleukin-17 (IL-17), and perhaps through direct cell–cell contact (60). This sequence of events is central to the “immunoporosis” concept, which proposes that activated T cells govern bone health, either directly or indirectly, through cytokine networks (60, 75). Aging and chronic antigenic stimulation, for instance from persistent viral infections, shift the CD8^+^ T-cell subset composition considerably. The age-dependent expansion of CD8^+^CD28⁻ T cells is especially striking. These cells produce pro-inflammatory cytokines and have been tied to several disorders with an inflammatory component, including atherosclerosis, dementia, and osteoporosis (65). It seems likely that, in chronic inflammatory diseases, prolonged antigenic stimulation similarly drives the clonal expansion of pro-inflammatory CD8^+^ T-cell subsets, such as effector/memory cells, and thus worsens bone destruction (65). Therapies for severe autoimmune disease, such as hematopoietic stem cell transplantation, also influence bone health. Immune reconstitution after transplantation carries a risk of osteoporosis. Rapid repopulation of effector/memory CD8^+^ T cells after transplant has been linked to disease relapse and to complications that include osteoporosis. The swiftly reconstituted CD8^+^ T-cell pool may have a particular functional status or cytokine profile that tips the balance of bone remodeling.

### 6.3 Emerging roles of CD8^+^ T cells in secondary osteoporosis

The sections above focus on primary osteoporosis—postmenopausal, age-related, and glucocorticoid-induced. However, CD8^+^ T cells also show up in secondary forms of the disease, although the evidence is thinner. When bones are unloaded (bed rest, spinal cord injury, spaceflight), they lose mass quickly. In hindlimb-unloaded mice, CD8^+^ T cells accumulate in the bone marrow and turn pro-inflammatory: more RANKL, less IFN-γ (76). This shift partly reflects reduced muscle-derived IL-33, much like in PMOP. Exercise (discussed in 6.1) counteracts this change. A negative feedback loop between osteoclasts and CD8^+^ T cells helps maintain skeletal-immune homeostasis, and unloading may disrupt this circuit (77). Type 2 diabetes raises fracture risk even when bone density looks normal. Chronic high blood sugar drives formation of advanced glycation end products (AGEs) and oxidative stress, which push CD8^+^ T cells toward senescence and a pro-inflammatory profile. In type 2 diabetes, senescent CD8^+^ T cells exhibit mitochondrial dysfunction and altered nutrient uptake, which drives their pro-inflammatory phenotype (44). Diabetic mice have more CD8^+^CD28⁻ T cells in the bone marrow. These CD8^+^CD28⁻ T cells inhibit osteoblast differentiation and reduce MSC survival, thereby contributing to bone loss (8) that correlates with fewer osteoblasts and more cortical porosity. Metformin, which improves T-cell metabolism, may partially reverse the damage. Rapid bone loss often follows hematopoietic stem cell or solid organ transplantation. Immune reconstitution after HSCT starts with a surge of effector memory CD8^+^ T cells (TEMRA). Expansion of CD28⁻CD8^+^ T cells after HSCT is associated with immune dysregulation and may contribute to bone loss (78), which are highly cytotoxic and carry granzyme B—potentially harming osteoprogenitors. Chronic graft-versus-host disease (cGVHD) worsens bone loss and comes with a lasting expansion of senescent CD8^+^ T cells. Current care leans on anti-resorptive drugs, but immune-based strategies aimed at CD8^+^ subsets are being explored. In short, CD8^+^ T-cell dysregulation appears across several types of secondary osteoporosis. Yet, each condition probably has its own distinct immune signature. We will need mechanism-driven studies tailored to each cause before any subset-specific therapy can be designed (**Table 3**).

**Table 3:** Therapeutic strategies targeting CD8^+^ T cells in osteoporosis.

Intervention	Specific approach	Mechanism on CD8^+^ T cells	Osteoprotective effect	Stage/evidence	Refs
Exercise	Wheel-running (8wk)	↑ splenic/bone marrow CD8^+^ T cells; restores IFN-γ	IFN-γ inhibits NF-κB/MAPK → ↓osteoclasts	Animal (OVX mouse)	(9)
Exercise	Weight-bearing/muscle strengthening	↑ muscle IL-33 → acts on ST2^+^CD8^+^ T cells	Modulates CCL5 → muscle-bone axis	Animal (rat)	(28)
Precision targeting	CCR3 antagonist (block CCL5/CCR3)	Blocks CD8^+^-derived CCL5 binding	Suppresses CCL5/CCR3/ERK osteoclastogenesis	Animal	(28)
Precision targeting	Anti-RANKL (denosumab)	Indirect modulation of T-cell responses	Inhibits osteoclast differentiation	Clinically approved	(4)
Natural product	Kaempferol (RA model)	Modulates NLRP3/CASP1/GSDMD; ↓CD4/CD8 ratio; ↓effector memory CD4^+^; ↑Treg	Suppresses overactive immunity; anti-pyroptosis	Network pharm + animal (CIA)	(80)
Natural product	Kaempferol (OP model)	Binds RPN1 (−7.2 kcal/mol); reverses high RPN1 in femur (RPN1 correlates with CD8^+^ T infiltration)	Improves bone microarchitecture	Bioinformatics + OVX rat	(73)
Cell therapy	HSCT	Reconstitutes balanced T-cell repertoire	Restores osteoimmune homeostasis (GVHD risk)	Case series	(82)
Metabolic intervention	Glutamine metabolism modulation	Improves CD8^+^ T-cell antitumor activity (indirect)	Restores immune function (inferred)	Tumor model	(48)
Immune checkpoint	PD-1/PD-L1 blockade	Expands bone marrow T-cell subsets; ↑ osteoclast activity (T-cell-dependent)	Bone loss (caution: context-dependent)	Animal	(81)

↑, Increase, upregulation, or enhancement (e.g., ↑ splenic CD8^+^ T cells); →, Leads to, results in, or triggers (e.g., “IFN‑γ → ↑NF‑κB/MAPK”).

## 7 Clinical translation: harnessing protective immunity while avoiding immunopathology

### 7.1 Exercise immunology: reprograming CD8^+^ T cells to preserve bone mass

Strategies aimed at boosting protective CD8^+^ T-cell responses show considerable promise, and exercise is the most clinically relevant and safest intervention for engaging this osteoprotective capacity. Unlike pharmacological immune activators, mechanical loading induces a context-dependent functional reprograming of CD8^+^ T cells that is both systemic and site-specific. The net effect is a shift in the osteoimmune balance toward bone formation, achieved without triggering generalized inflammation. Two interconnected pathways account for this protection. Exercise directly augments the production of interferon-γ (IFN-γ) by CD8^+^ T cells. In ovariectomized (OVX) mice, an 8-week wheel-running protocol increased the proportion and absolute number of IFN-γ-secreting CD8^+^ T cells in both spleen and bone marrow (9). The resulting elevation in IFN-γ exerts a direct inhibitory effect on osteoclast precursors. It accelerates TRAF6 degradation and thereby dampens the NF-κB and MAPK signaling cascades that are essential for osteoclast differentiation (10). This immunomodulatory effect is dose- and context-dependent, a feature that deserves emphasis. Physiological levels of IFN-γ generated by exercise restrain resorption, whereas pathologically high concentrations (as can occur in chronic inflammation) paradoxically promote bone loss. The narrow therapeutic window explains why endogenous regulation via exercise is safer than exogenous cytokine administration. A second mechanism has emerged from recent work on muscle–bone crosstalk, centered on the myokine IL-33. Skeletal muscle contraction releases IL-33, which acts on ST2^+^ bone marrow CD8^+^ T cells to suppress their secretion of the chemokine CCL5 (28). In sedentary or sarcopenic states, reduced IL-33 tone unleashes CCL5 production, and the resulting CCL5/CCR3/ERK signaling drives osteoclastogenesis. Weight-bearing exercise restores muscle-derived IL-33, normalizes the CD8^+^ T-cell secretory profile, and interrupts a key pathological axis in postmenopausal bone loss. From a translational standpoint, these data support the view of exercise as a form of *in vivo* cellular therapy for CD8^+^ T cells. The bone-protective effects of IFN-γ and the suppression of CCL5 are most pronounced with moderate-intensity, long-duration exercise. Exhaustive or eccentric protocols may induce transient immunosuppression or elevate pro-inflammatory cytokines and are therefore less suitable. For clinical practice, the recommendation is clear: consistent weight-bearing aerobic activity (brisk walking, jogging) should be combined with progressive resistance training to maintain muscle mass and IL-33 secretion. A note of caution applies to frail elderly patients with high baseline inflammation. In this population, the initial phase of exercise may transiently activate pro-inflammatory CD8^+^ T-cell subsets. A gradual progression of intensity is therefore advised to allow the osteoimmune microenvironment to adapt (**Table 4**).

**Table 4:** Heterogeneity of CD8^+^ T-cell subsets and relevance to osteoporosis.

CD8^+^ T-cell subset	Phenotypic markers	Functional characteristics	Potential role in osteoporosis	Refs
Naïve	CD45RA^+^CCR7^+^	High plasticity, antigen-inexperienced	Primary response to bone microenvironment changes	(83–85)
TCM	CD45RO^+^CCR7^+^	Long-lived, rapid recall, LN homing	Positively correlated with high BMD	(45, 83)
TEM	CD45RO^+^CCR7⁻	Rapid effector, tissue homing	KLRB1^+^IL7R^+^ in SSc; enhanced cytotoxicity → tissue damage	(79, 86)
TEMRA	CD45RA^+^CCR7⁻	Potent cytotoxicity, short telomeres, CD28 loss	Negatively correlates with fracture healing; TEMRA/Treg imbalance prognostic	(20, 34)
Senescent CD8^+^	CD28⁻CD57^+^	Loss of CD28, reduced proliferation	Inhibits osteoblast differentiation and MSC survival	(8, 37)
TcREG	CD8^+^FoxP3^+^CD25^+^	Immunosuppressive; express T-bet, Eomes, IFN-γ	Suppresses bone resorption via TRAF6 degradation	(10)
CD8a^+^GZMK^+^	CD8a^+^GZMK^+^GZMA↓	Secrete granzyme K	Inhibits osteoclastogenesis via p38-MAPK; negatively correlates with bone loss	(12)

### 7.2 Precision targeting of the CCL5/CCR3 axis: a new frontier for immunotherapy in osteoporosis

The identification of CD8^+^ T cells as bifunctional regulators of bone mass creates both an opportunity and a warning for clinical practice. Exercise harnesses protective pathways, but pharmacological neutralization of specific pathogenic factors offers an even more precise immunoregulatory approach. The chemokine CCL5 (RANTES), secreted by CD8^+^ T cells, has emerged as a non-redundant driver of bone loss in preclinical models of postmenopausal osteoporosis. Upon binding to the CCR3 receptor on osteoclast precursors, CCL5 activates the ERK pathway and thereby promotes both the differentiation and the resorptive activity of mature osteoclasts (28). This pathway operates downstream of estrogen withdrawal and muscle atrophy, yet upstream of RANKL signaling. CCL5 blockade could therefore synergize with, or provide an alternative to, conventional anti-resorptive agents such as bisphosphonates or denosumab. Moreover, CCL5 secretion is largely confined to activated CD8^+^ T cells within the bone marrow niche. Targeting this axis may thus permit site-specific immunomodulation without systemic immunosuppression. Preclinical evidence supports the viability of this strategy. In OVX mice, pharmacological blockade of CCR3 (using small-molecule antagonists) phenocopies the bone-protective effects of exercise and IL-33 administration. Trabecular bone volume fraction is preserved, and osteoclast surface is markedly reduced (28). Monoclonal antibodies against CCL5, originally developed for oncology or HIV-related inflammation, could in principle be repurposed for osteoporosis. Early-phase studies in other disease settings have reported acceptable safety profiles with respect to bone endpoints, although dedicated skeletal imaging was rarely included. Several translational hurdles remain. A key barrier is biomarker development. No validated clinical assay currently exists to quantify bone marrow CD8^+^ T cell-derived CCL5. Peripheral blood levels are confounded by platelet contamination and systemic inflammation and therefore offer limited utility as a surrogate. The development of flow cytometric assays for ST2^+^/CCL5^+^ CD8^+^ T cells in bone marrow aspirates may prove necessary for patient stratification. Safety considerations also deserve scrutiny. CCL5 is a pleiotropic chemokine that participates in antiviral immunity and leukocyte trafficking. Transient or local blockade is well-tolerated in animal models, yet chronic systemic neutralization could theoretically impair immune surveillance. Intermittent dosing regimens or bone-targeted delivery systems (for example, bisphosphonate-conjugated antibodies) represent attractive ways to maximize skeletal benefit while containing systemic immunological risk.

### 7.3 Immunomodulatory agents in osteoporosis: navigating the double-edged sword

Indiscriminate activation of T-cell immunity carries substantial risks for skeletal integrity. A clear distinction must therefore be drawn between osteoprotective immunomodulation and high-risk immune activation. The broader landscape of immunomodulatory drugs makes this dichotomy explicit. Agents that restore or mimic the physiological restraint exerted by CD8^+^ T cells (exercise, IL-33 mimetics) are osteoprotective. Agents that cause broad-spectrum T-cell activation or cytokine release, in contrast, carry a high risk of accelerating bone resorption. Several natural compounds, including kaempferol, can modulate CD8^+^ T-cell infiltration and activation status in inflammatory models of arthritis and osteoporosis (73, 80). These compounds typically have a favorable safety profile. Their mechanisms, however, are polypharmacological and rarely specific to a single CD8^+^ T-cell pathway. Their clinical utility in osteoporosis may be greatest as adjunctive therapy (for instance, in patients with mild inflammatory comorbidities) rather than as primary anti-resorptive agents. Rigorous randomized controlled trials with bone mineral density as a primary endpoint are still lacking. A critical distinction exists between modulating CD8^+^ T-cell function (as with exercise or CCL5 blockade) and activating it indiscriminately. Immune checkpoint inhibitors (ICIs), such as anti-PD-1 and anti-CTLA-4 antibodies, exemplify the dangers of high-risk immune activation for the skeleton. Preclinical studies indicate that PD-1/PD-L1 blockade expands bone marrow T-cell subsets and enhances osteoclast activity in a T cell-dependent manner (81). Emerging clinical data further link ICI therapy to accelerated bone loss and increased fracture risk, particularly in cancer patients receiving combination immunotherapy. Clinical alert: Patients who receive ICIs for malignancy should undergo baseline bone density assessment and proactive management of bone health. Early use of anti-resorptive agents (bisphosphonates or denosumab) should be considered, because the pro-osteoclastic drive from activated CD8^+^ T cells may blunt the efficacy of lifestyle interventions alone. Hematopoietic stem cell transplantation (HSCT) leads to immune reconstitution that can affect bone health, and rapid expansion of certain CD8^+^ T-cell subsets has been linked to complications including osteoporosis (82). In this specific form of secondary osteoporosis, management should prioritize control of GVHD and include standard anti-osteoporotic pharmacotherapy. HSCT is not a viable strategy for primary osteoporosis; it is mentioned here only to illustrate the skeletal consequences that can follow a shift in the CD8^+^ T-cell repertoire.

## 8 Research challenges and future perspectives

### 8.1 Deeper investigation of CD8^+^ T-cell heterogeneity and functional plasticity

CD8^+^ T cells are far from a uniform population. They encompass several functional subsets—naïve, central memory (TCM), effector memory (TEM), terminally differentiated effector, and tissue-resident memory (TRM) cells—that play distinct roles in immune responses (83–85). Under chronic pathological conditions such as osteoporosis, the contributions of different subsets are likely to differ considerably. In the skin lesions of systemic sclerosis, for instance, effector memory CD8^+^ T cells (KLRB1^+^IL7R^+^) display enhanced cytotoxicity and Tc2/Tc17 effector functions, features that may drive tissue damage and fibrosis (86). Exhausted CD8^+^ T cells (KLRG1^+^IL7R⁻), on the other hand, exhibit a transcriptional signature characteristic of long-term effector cells and may promote chronic inflammation (79, 87). These observations raise the possibility that specific CD8^+^ T-cell subsets within the bone microenvironment participate in bone destruction or sustain chronic inflammation through similar mechanisms. High-throughput techniques such as single-cell RNA sequencing (scRNA-seq) can resolve the transcriptomic and proteomic profiles of individual CD8^+^ T-cell subsets in the bone microenvironment with precision. This capability is essential for deciphering their specific roles in osteoporosis (88, 89). In chronic infection, scRNA-seq has revealed that exhausted CD8^+^ T cells possess organ-specific transcriptomes and can be further divided into five major functional subpopulations (88, 90). Similarly, in lung adenocarcinoma, scRNA-seq analysis has uncovered complex differentiation trajectories and heterogeneity among infiltrating CD8^+^ T cells, spanning naïve-like states through to cytotoxic and exhausted phenotypes (91, 92). These approaches are equally applicable to studies of CD8^+^ T-cell subset dynamics and their regulatory determinants under different pathological conditions, including aging and estrogen deficiency. The naïve CD8^+^ T-cell pool itself exhibits functional heterogeneity and plasticity. Certain subpopulations, such as those expressing IL-18Rα, CD73, CXCR3, or Dapl1, have an intrinsic propensity to differentiate into high-quality effector and memory cells (93). During aging, CD8^+^ T-cell heterogeneity and the transcriptome undergo marked changes, and subset dynamics correlate closely with differentiation state and mutational burden (93, 94). Furthermore, the transcriptional cofactor Tle3 dynamically governs the fate choice and lineage stability of effector memory versus central memory CD8^+^ T cells by integrating the activities of multiple transcription factors, including T-bet, Runx3, and Tcf1 (95). A deeper exploration of this inherent heterogeneity, plasticity, and the underlying regulatory networks will help to identify more specific interventional targets. Modulating particular transcription factors or epigenetic modifications to shape the functional state of CD8^+^ T cells could, for example, open new avenues for immunotherapy in osteoporosis (96, 97).

### 8.2 Cross-organ communication and a systems biology perspective

The immunoregulatory reach of CD8^+^ T cells goes well beyond the local bone microenvironment and includes complex cross-organ communication. A systemic view of osteoporosis pathogenesis is therefore needed. The IL-33–CCL5 axis uncovered in muscle–bone crosstalk, for example, points to a central role for immune cells in interorgan signaling (28). Other axes, including the gut microbiota–immune–bone axis and the adipose tissue–immune–bone axis, also contribute to the systemic governance of bone metabolism (98). In inflammatory bowel disease, CD8^+^ T-cell heterogeneity and functional plasticity are tightly linked to disease initiation and progression, which implies that intestinal immune status may shape bone metabolism through systemic inflammation (99, 100). In tuberculous pleurisy, tissue-resident memory-like CD8^+^ T cells are phenotypically and functionally diverse, and local cues such as TGF-β regulate their differentiation (101). These findings show that the resident tissue microenvironment strongly shapes CD8^+^ T-cell function and that immune signals exchanged between organ systems probably influence skeletal homeostasis in a collective manner (102). Future work should integrate metabolomic, microbiome, and immunological data to build network models of multiorgan interaction. Such models are essential for a comprehensive view of where CD8^+^ T cells sit within the systemic control of bone metabolism. Single-cell sequencing and spatial transcriptomics can reveal the spatial organization and heterogeneity of T-cell subsets within tissues and map their crosstalk with the local matrix (103). In tumor immunology, a holistic analysis of monocyte-to-macrophage differentiation has linked Treg abundance to CD8^+^ T-cell functional status, illustrating the interdependence of cell types within the immune ecosystem (104). In type 2 diabetes, single-cell transcriptomics has captured a shift in CD8^+^ T cells from a pro-angiogenic tissue-resident memory state toward effector and effector memory fates, a plasticity that directly controls vascular regeneration (105). This work offers a paradigm for understanding CD8^+^ T-cell contributions to bone loss in metabolic disease. To grasp the systemic nature of osteoporosis, it is essential to learn how CD8^+^ T cells interpret signals from distant organs—the gut, fat, or muscle—and translate them into local effects on osteoclasts or osteoblasts. Multi-omics systems models will help to identify the core pathways and hub molecules that drive osteoimmune imbalance and will support the design of interventions that target interorgan crosstalk (106).

### 8.3 Clinical translation and precision medicine

Much of what is known about CD8^+^ T cells in osteoporosis comes from animal models and *in vitro* work. Well-designed clinical studies are now required to move these findings into the clinic. A key early step is to verify that specific CD8^+^ T-cell subsets, or cytokines such as CCL5 and IFN-γ, can serve as biomarkers for diagnosis, prognosis, or the prediction of treatment response. In cancer immunotherapy, particular intratumoral CD8^+^ T-cell subsets (for instance, TCF1^+^ progenitor-like exhausted precursors) and their transcriptional profiles track closely with patient responses to PD-1 blockade (107, 108). A similar approach in osteoporosis, profiling the distribution of CD8^+^ T-cell subsets (TEMRA, TCM, TRM, and exhausted cells) in peripheral blood or bone tissue by flow cytometry or single-cell sequencing, may reveal immune signatures linked to bone mineral density, fracture risk, or disease activity (109, 110). Immune phenotyping that reflects CD8^+^ T-cell functional status could allow finer stratification of patients. Individuals might be grouped according to whether they harbor high levels of pro-inflammatory CD8^+^ T-cell subsets (such as Tc1 or Tc17) or whether dysfunctional exhausted subsets predominate (99, 107, 111). This classification would help tailor immunomodulatory therapy. Patients in whom overactive CD8^+^ T cells drive bone destruction might receive cytokine inhibitors, for example anti-IFN-γ or anti-CCL5 antibodies (112). Those with signs of T-cell exhaustion or senescence, by contrast, could benefit from immune-enhancing agents (e.g., IL-7 or IL-15) or metabolic interventions that aim to restore T-cell function, including the modulation of glutamine metabolism or mitochondrial health (113, 114). Lifestyle measures, such as dietary changes (sodium intake can affect CD8^+^ T-cell effector function) and exercise, may also improve systemic immune status (115, 116). Experience in cancer immunotherapy suggests that targeting immune checkpoints like PD-1 is promising for reversing T-cell exhaustion. The safety and efficacy of such strategies in osteoporosis still require careful testing (81, 107). Predictive models that combine clinical data, imaging metrics, and high-dimensional immune profiles will eventually support more accurate risk assessment and treatment decisions. This integration should push osteoimmunology closer to genuinely personalized care (106, 117).

### 8.4 A key limitation: interactions with other immune cells

So far, we have focused on what CD8^+^ T cells do alone. However, in the bone marrow, they live in a crowded neighborhood. They talk to—and listen to—other immune cells all the time: CD4^+^ T cells, regulatory T cells (Tregs), B cells, macrophages, and others. These interactions can flip the net effect on bone (118, 119). CD8^+^ T cells can steer CD4^+^ T helper differentiation by releasing cytokines. For example, IFN-γ from CD8^+^ cells pushes Th1 polarization (60). That shift may then affect osteoclastogenesis through IL-12 and more IFN-γ (60). On the other side, IL-17 from CD4^+^ cells can activate CD8^+^ T cells, creating a pro-inflammatory loop (60, 77). The well-known Th17/Treg balance—a key driver of bone loss—is partly regulated by CD8^+^ T cell-derived IL-10 and TGF-β (77, 120). Macrophages are a major source of TNF-α and IL-6, two potent osteoclast-forming cytokines. Activated CD8^+^ T cells recruit macrophages and push them toward an M1 (pro-inflammatory) state, using IFN-γ and TNF-α (1). Those M1 macrophages then secrete IL-12 and IL-18, which further activate CD8^+^ T cells (1). This positive feedback loop amplifies bone resorption (1). Conversely, CD8^+^ Treg cells may promote M2 macrophages, which help bone formation (1). B cells produce RANKL and play a real role in bone metabolism. Estrogen deficiency increases B-cell numbers and their RANKL expression (121). CD8^+^ T cells can suppress B-cell activation and antibody production via IFN-γ and direct contact. However, the overall effect on bone is messy—it depends on context. In rheumatoid arthritis, CD8^+^ T cells and B cells cooperate inside ectopic lymphoid structures to drive local bone erosion (122). Most studies—including our own summary—have examined CD8^+^ T cells in isolation or in simple two-cell cocultures. That does not reflect the real complexity of the bone marrow immune niche (123). Going forward, we need high-dimensional tools: single-cell RNA-seq, multiplex imaging, and *in vivo* lineage tracing (123). Only then can we map the entire immune network at once. Until we know whether CD8^+^ T cells are master regulators or just one node among many, any therapy that targets them alone should be approached with caution. Unintended side effects on other immune populations are likely.

## 9 Conclusions

CD8^+^ T cells occupy a complex and central position in osteoimmune homeostasis. Their role now goes well beyond classical cytotoxicity. They function as dynamic, tightly regulated modulators of bone metabolism that respond to multiple signaling networks. Under physiological conditions, CD8^+^ T cells help restrain osteoclast formation, chiefly through the secretion of factors such as IFN-γ, and thus act as essential guardians of bone mass. In pathological settings (postmenopausal osteoporosis, aging, and various forms of secondary osteoporosis), this functional state shifts markedly. The change does not stem from a single cause. It involves a convergence of interconnected processes: activation of NF-κB signaling after estrogen withdrawal, accumulation of senescent CD8^+^CD28⁻ T cells that release a senescence-associated secretory phenotype (SASP), and intrinsic metabolic reprogramming that includes altered energy metabolism and endoplasmic reticulum stress. Together, these events turn CD8^+^ T cells from protectors of bone into important sources of pro-osteoclastogenic factors, most notably CCL5, and thereby drive bone loss. The actions of CD8^+^ T cells are highly context-dependent and heterogeneous. Different etiologies (estrogen deficiency, glucocorticoid use, or chronic inflammation) may affect specific CD8^+^ T-cell subsets through distinct or overlapping routes and produce characteristic shifts in their functional repertoire. Because of this variation, the net contribution of CD8^+^ T cells differs across osteoporotic models and clinical scenarios. Current research is centered on this challenge. Single-cell sequencing, spatial transcriptomics, and related technologies now allow a detailed look at CD8^+^ T-cell heterogeneity within the bone microenvironment. The aim is to define subsets that promote bone resorption versus those that preserve bone mass, and to map their surface markers, transcriptional signatures, and operative pathways. This mechanistic picture supports a growing interest in CD8^+^ T cell-directed interventions. The benefits of exercise, certain immunomodulatory drugs, and natural products can be traced, at least in part, to favorable effects on CD8^+^ T-cell abundance, activation state, senescence, or secretory profile, including the suppression of pro-osteoclastogenic factors such as CCL5. More precise strategies that target key signaling axes, for instance the IL-33–ST2–CCL5–CCR3 cascade, suggest a new generation of targeted therapies. A quicker transition from mechanism to clinical application is now a priority. A clearer view of how distinct CD8^+^ T-cell subsets behave during the onset and progression of human osteoporosis would help to identify reliable biomarkers for early diagnosis, risk stratification, and treatment monitoring. At the same time, strategies aimed at specific CD8^+^ T-cell subsets or pathways need to be developed into safe, effective clinical regimens, whether through selective antibodies, small-molecule inhibitors, or cell-based approaches. A deeper integration of bone biology and immunology, built on a refined understanding of the CD8^+^ T-cell regulatory network, should open new and more targeted immunotherapeutic avenues for osteoporosis. Granzyme K has emerged as a previously overlooked inhibitor of osteoclastogenesis, and the IL-33–ST2 axis now appears to coordinate crosstalk across muscle, bone, and the immune system. What distinguishes the two major disease pathways is this: senile osteoporosis is driven largely by dysfunctional immunosenescence—marked by CD28⁻CD8^+^ T cells and their SASP—whereas postmenopausal osteoporosis (PMOP) involves a more inflammatory T-cell skewing. Future precision medicine will need to stratify patients by these immuno-endotypes rather than by bone density alone. Boosting IL-33 signaling, for example, might counteract its age-related decline, whereas clearing senescent CD8^+^ T cells with senolytics could serve a dual purpose: curbing pathological bone resorption and, at the same time, supporting formation.

## Data Availability

The datasets presented in this study can be found in online repositories. The names of the repository/repositories and accession number(s) can be found in the article/supplementary material.
